# Finite Element Simulation Tests of the Structural Strength of the Molding Module for Burger Production from Vegetable Outgrades

**DOI:** 10.3390/ma14226747

**Published:** 2021-11-09

**Authors:** Łukasz Ignasiak, Agata Bieńczak, Paweł Woźniak, Katarzyna Kozłowicz, Renata Różyło, Jan Szczepaniak

**Affiliations:** 1 Łukasiewicz Research Network-Industrial Institute of Agricultural Engineering, Starołęcka St. 31, 60-963 Poznan, Poland; lukasz.ignasiak@pimr.lukasiewicz.gov.pl (Ł.I.); agata.bienczak@pimr.lukasiewicz.gov.pl (A.B.); pawel.wozniak@pimr.lukasiewicz.gov.pl (P.W.); jan.szczepaniak@pimr.lukasiewicz.gov.pl (J.S.); 2 Department of Biological Basis of Food and Feed Technology, University of Life Sciences in Lublin, Głęboka St. 28, 20-950 Lublin, Poland; katarzyna.kozlowicz@up.lublin.pl; 3 Department of Food Engineering and Machines, University of Life Sciences in Lublin, Głęboka St. 28, 20-950 Lublin, Poland

**Keywords:** vegetable waste, burgers, molding module, finite element simulation

## Abstract

The aim of the study was to assess the stresses of the structural materials of the forming module in the process of burger production from vegetable outgrades. The simulation research object was a virtual CAD 3D model of a device used for forming multi-vegetable products. Strength tests were performed on the computational model by applying the finite element method. The following were analyzed in the model: the forces exerted by the mixture of vegetables on the side walls of the tank and the dosing unit; the force from the servomotor resulting from the horizontal thickening of the vegetable mixture; the force from the servomotor resulting from the vertical mixing of the vegetable mixture; the force from the die assembly actuator; the force caused by punching the actuator from the die assembly. For evaluating the structure in the scope of the study, it was assumed that safely reduced stresses should be taken into account, with a safety factor equal to 1.1 of the yield strength of the parent material from which the structure was made (steel 1.4301 (304) with a yield stress Re_0.2_ of 230 MPa). For welds, safely reduced stresses should be taken into account, with a safety factor equal to 1.4 of the yield strength (Re_0.2_ of 230 MPa). Strength analyses confirmed that the permissible stress levels were not exceeded in the molding module.

## 1. Introduction

Food waste has a significant negative impact on the economy, environment, and society [1], and mainly concerns highly developed countries [2,3,4]. It has been identified throughout the supply chain from the direct consumer to sales, transport, and manufacturing processes, or throughout the process of food production “from farm to fork”. According to the expert opinions prepared by the Analysis and Thematic Studies Team of the Analysis and Documentation Office in April 2016 on “How to avoid food waste—strategies to improve the efficiency of the EU distribution chain in food donation” [5], according to Eurostat estimates, food waste is generated in 27 EU countries. Much of the food waste comes from households [1,6], accounting for as much as 42%. The second-largest amount of food waste occurs in production [5]. Food production processes generate a large quantity of waste, which is often nutrient-rich. Although there is a trend towards using waste to produce valuable products, most of the generated waste is still disposed of [7,8]. Unfortunately, no information is available regarding the stage of food production that gives rise to most of the waste. Production plants are not able to manage products considered as post-production waste, such as the waste (outgrades) of vegetables resulting from selection during the manufacture of frozen food. These vegetables usually do not meet the standards relating to size and shape and hence are considered waste. However, such vegetable waste is a valuable food product, as it contains bioactive compounds, enzymes, and nutrients [9]. Currently, vegetable waste is used in the production of multi-vegetable soup mixes. Nevertheless, the supply of vegetables exceeds the demand for the finished product, and hence, vegetable waste management is recognized as a serious problem.

Studies have been carried out to develop a technology for managing waste resulting from the production of multi-vegetable burgers. In Łukasiewicz Research Network–IIAE, a processing line for burger production from vegetable waste has been developed. The most important goal of this processing line is to achieve the proper shape of burgers. In the design process, it was considered important to determine the state of effort of the construction material via computer mechanics using finite element methods (FEMs), which are mathematical methods for physical calculations and involve replacing a real object with elements of finite dimensions, averaging its physical state [10].

Finite element modeling can potentially be used in many experimental studies. At present, it is widely applied in various branches of medicine [11,12,13], including orthopedics, cardiovascular surgery, and dentistry [13]. For considering food-grade applications, a high-pressure extractor was designed and analyzed using FEA. In these studies, a special clamping system was designed for an extractor and was analyzed using FEA [14]. Another study optimized the design of an ultrasonic transducer for food dehydration using a particle swarm optimization (PSO) algorithm and finite element analysis (FEA) [15]. Another paper presented a design analysis of rotary turret plates for food product packaging machines, also using FEA analysis [16]. Strength studies on the screw assembly of food screw presses were also performed, based on large-scale FEA. The authors considered that this study could provide a certain theoretical basis and basic methods for designing a more stable screw press [17].

FEM aids in solving strength-related problems in the field of mechanical engineering [18,19,20] and in studying structures with complex physical properties [21,22,23]. The elastomechanical characteristics of forming machines are a critical element in various forming processes. To understand the complex interactions between the process, forming tool, and machine, it is essential to carry out numerical simulations [24]. The use of FEM allows the generation of discrete triaxial fields and determination of the necessary local thickening. This method can be used to produce functional prototypes at an early stage of design [25]. For developing computational models, mechanical structures with complex shapes are subjected to geometric simplifications. During simplification, the geometry of three-dimensional (3D) elements with a complex shape is simplified, 3D elements with a less complex shape are replaced by two-dimensional (2D) plate-shell elements, structural connections are created using one-dimensional (1D) elements (nodal connections), and additional mass loads are reduced to a concentrated mass (located at a point). All these modifications make the engineering analyses more efficient. The simplified model is subjected to discretization, during which it is divided into a finite number of 2D elements (rectangular or triangular) or 3D elements (tetrahedral). Ultimately, the boundary conditions for obtaining a solution are defined. As a result, different values are determined for individual interconnected nodal finite elements, for example, stresses and displacements in the case of static analyses and, additionally, accelerations and velocities for dynamic analyses. The data obtained from the analyses enable the strength and safety of the structure to be inferred and serve as guidelines for introducing structural changes [23].

The finite element methodology has been utilized to calculate the deflections of example composite structures [26]. There are very few articles in the literature regarding finite element simulation of molding machines. In a previous study, a mechanism injecting molten plastic into a mold was simulated, to study the fatigue degradation of some parts of the molding machine. The work described the finite element analysis of the fatigue failure of a piston screw (i.e., a screw pump) [27]. Another study analyzed why and how machine rigidity affects the final thickness of formed glass rings. Finite element simulations were performed, combined with thermal displacement, to demonstrate how forming behavior and thickness evolution are influenced by machine stiffness. The results of these studies showed that the final thickness deviation is mainly related to the maximum deformation of the machine during pressing, and thus decreases with machine stiffness. The effect of machine stiffness decreases markedly with forming temperature, which also explains the decrease in the final thickness in experimental results [28]. Another study performed finite element simulation to determine the structural strength of a molding machine employing pneumatic clay stamping. The tests conducted in the study also aimed to identify the most vulnerable locations in the machine structure that may cause a problem when the load capacity is increased [29].

The finite element method used for strength analyses is a method known and used in the design of machines and devices. A novelty in this article is the development of forming device design solutions which have been subjected to strength analyses in virtual computer models. 

## 2. Materials and Methods

**Figure 1:**
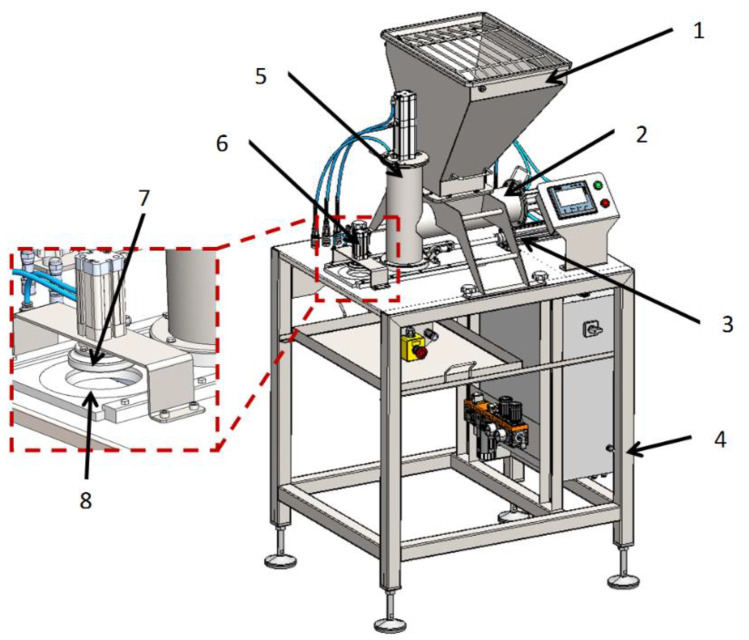
Virtual model of the forming module: 1. technological tank; 2. horizontal dispenser with an actuator for thickening the vegetable mixture; 3. actuator of the die assembly extension; 4. support frame (table); 5. vertical dispenser with an actuator for thickening the vegetable mixture; 6. actuator ejector; 7. ejector; 8. die assembly.

The simulation test object was a virtual CAD 3D model of a molding machine used to shape burgers. The process of vegetable burger preparation begins in the molding module (patent application no. P.438481) (Figure 1). During the process, the vegetable mixture is collected in the technological tank, and then moved to the die assembly using dispensers with pushers and pneumatic actuators (forming the molding center). The reciprocating movement is performed by an actuator in the die assembly. As the die assembly is extended, the ejector actuator knocks the formed veggie burger out of the die, and the burger finally falls onto the conveyor.

The construction of the computational model of the molding module, as well as the multivariate structural analyses, were carried out in the NX system. The following elements were used to build the model:

(a) One dimension:
  - beam elements of the BEAM type and rod elements of the ROD type for modeling pneumatic cylinders and
  - RBE2 rigid elements for modeling point welds and other connections that facilitate load transfer between elements;
(b) Two dimensions: plate-shell modeling of the surface of sheet metal structures;
(c) Three dimensions: tetrahedral [30] (tetrahedral) finite elements for modeling the sliding elements, die assembly, dispenser runners, and pushers.

**Figure 2:**
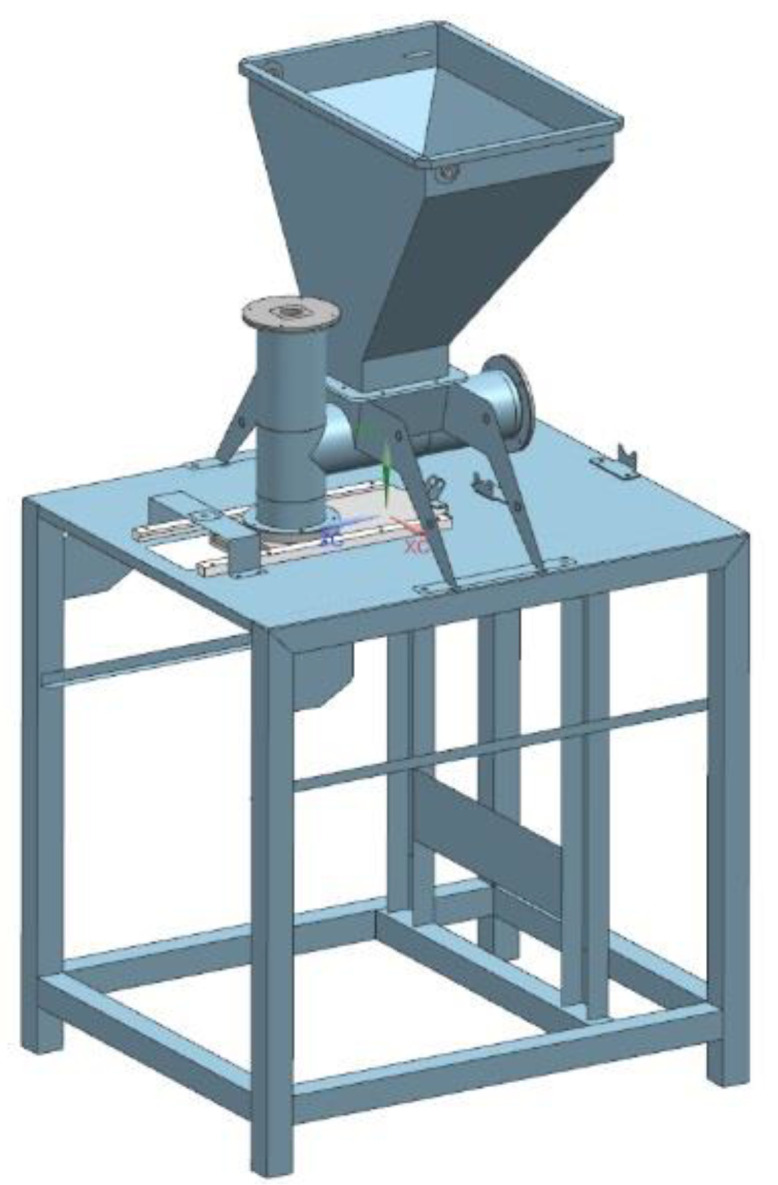
Plate-shell model of a forming module with solid elements.

The solid sheet elements and sections were converted into plate-shell elements, whereas the die assembly and dispenser pushers were left as solid elements during simplification. The simplified plate-shell models with solid elements are shown in Figure 2.

This paper presents static calculations applied for loads arising in the forming module during normal operation in the given range, as well as for loads arising during undesirable situations (e.g., in the event of a collision of the ejector with the die assembly). 

The object was not analyzed in terms of dynamics. It was decided that static analysis would be fully sufficient for the considered structure, for the purpose of building a prototype and carrying out further experimental research. The static analysis took into account the maximum possible forces acting in the system. Extreme cases that may occur during the operation of the device were considered.

After taking into account the force generated by the 800 N actuator, the value of which was determined based on the maximum force that can be generated by a pneumatic actuator with a piston diameter of 40 mm and a supply pressure of 6 bar, the following design cases were considered:

1. Blockage of the horizontal dispenser pusher against the vegetable mixture accumulated in the chamber and the resulting pressure on the vegetable mixture;
2. Blockage of the vertical dispenser pusher against the vegetable mixture accumulated in the chamber and the resulting pressure on the vegetable mixture;
3. Locking of the die assembly in the runners during an attempt to shift the die;
4. Accidental collision of the ejector with the die assembly, with the force generated by the actuator amounting to 300 N for a piston diameter of 25 mm.

The final force pattern on the surface of the actuator piston during the stroke of the piston rod takes the form [31]:

$$F=PA=P\left(\frac{\pi D^{2}}{4}\right)\;.$$

where:

*P* is the working pressure (Pa);
*F* is the thrust force (N);
*A* is the surface area (m^2^);
*D* is the piston diameter (m).

The results were assessed based on the equivalent stresses estimated using the Huber–Mises–Hencky (H–M–H) strength hypothesis or the shear energy hypothesis [32]. It is the most commonly used hypothesis for determining the achievement of the critical phase for complex stress states. 

### 2.1. Assumptions of Safety Indicators

For the assessment of the structure in the scope of this study, the equivalent stress, taking into account the safety factor, was equal to 1.1 of the yield strength of the material from which the structures are made. Since the material used was 1.4301 (304) steel having a yield stress Re_0.2_ of 230 MPa, the equivalent stress was σ_red_ ≤ 207 MPa. For the welds, the equivalent stress, due to the safety factor of 1.4 of the yield point, was σ_red_weld_ ≤ 138 MPa.

The structural steel used was 1.4301 steel, but there were also elements made from POM-C (polyacetal) plastic with a yield point of 67 MPa. Thus, the calculation model also took into account the material properties of POM-C for elements made from plastic, and the determined stress values in these elements were related to the permissible stress values provided for this material. Due to the low values of stresses in elements made from plastic, a detailed presentation of these results was omitted in this study. In the entire calculation model, friction was neglected.

### 2.2. Calculation Model of the Molding Module

**Figure 3:**
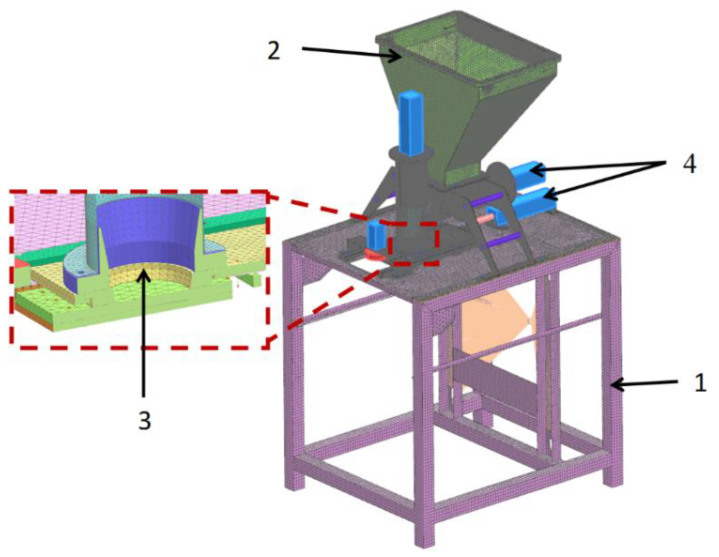
Calculation model of molding module: 1. support frame; 2. molding center with processing tank; 3. die assembly; 4. set of actuators.

The computational model of the molding module is shown in Figure 3. The molding center with the technological tank was located on a support frame, which was modeled with 1D, 2D, and 3D elements for strength analyses [33,34,35]. 

For some components, 1D and 2D elements were used to reduce the cost of simulation calculations. For example, by using 2D elements in the technological tank itself, we obtained 12.510 nodes, while when using 3D elements, we obtained 24.525 with a comparable mesh density. Simplifying the model by using 2D elements significantly reduces the number of nodes analyzed during the simulation and contributes to shortening the computation time.

**Figure 4:**
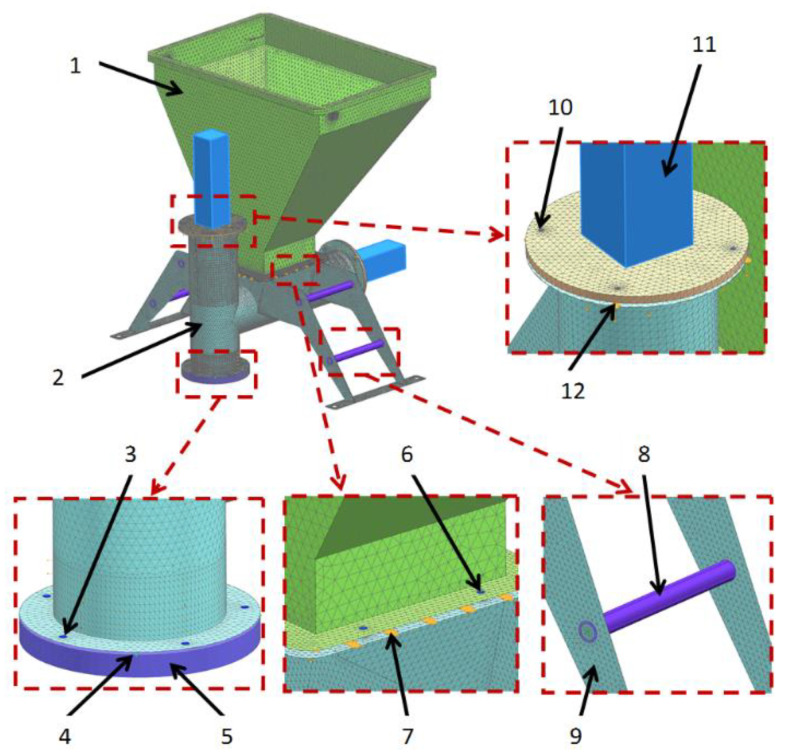
Calculation model of molding center with a processing tank. View 1: actuator, bolted connection, contact set, axis, and element (el.). 1. Technological tank (2D elements), 2. Vertical dispenser (2D elements), 3. Screw connection (1D elements), 4. Contact set, 5. Sliding unit (3D elements). 6. Screw connection (1D elements), 7. Contact set, 8. Connector (1D elements), 9. Forming center support brackets (2D elements), 10. Screw connection (1D elements), 11. Pneumatic actuator (1D elements), 12. Contact set.

**Figure 5:**
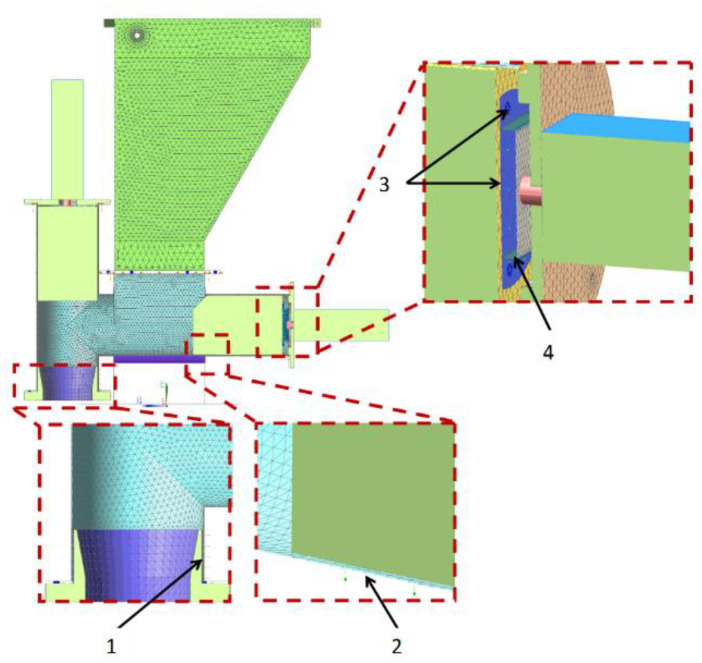
Calculation model of molding center with a processing tank. View 2: connection of the actuator with the pusher of the dispenser, bolted connection, contact set, piston rod, and rigid connection. 1. Contact set, 2. Contact set, 3. Bolted connection and contact set, 4. RBE2 rigid elements.

The computational model consisting of a forming center with a technological reservoir is shown in Figure 4 and Figure 5. The sheet metal structure and the pipes of the vertical and horizontal dispensers were modeled with 2D elements, while the pushers of the dispensers and flanges mounting the actuator were modeled with 3D elements. The axes connecting the supports of the entire assembly were modeled with 1D elements. In the places of contact of surfaces (non-welded elements), contact sets of the “surface–surface contact” or “surface–surface bonding” types were used. On the other hand, the connection points of the pneumatic cylinders with mounting flanges with RBE2 rigid elements were modeled using 1D elements. The areas A, B, C, and D define the most important parts of the computational model.

**Figure 6:**
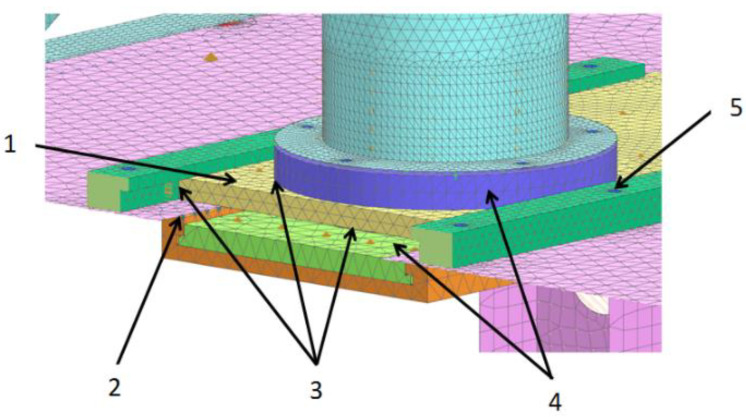
Calculation model of molding module connecting the die assembly with the molding center and table (runner rail: bolted connection, sliding elements, contact set, RBE2 rigid elements, and matrix assembly) and modeling of the interconnection of the die assembly with the forming center and table. 1. Die assembly, 2. RBE2 rigid elements, 3. Contact set, 4. Sliding elements, 5. Guide screw connection (1D elements).

The method of modeling the interconnection of the die assembly to the forming center and table is shown in Figure 6. The die assembly was able to slide over the sliding elements; therefore, the contact between these elements was modeled. The sliding elements, die assembly, and runners were modeled with 3D elements. Tetrahedral elements used in the computational model in 3D subassemblies were used to map the load transfer of mutually cooperating elements by giving them contacts both on the surface and on the side walls. The type of 3D element used was only used to evaluate the stress distribution, indicating places with the highest stress concentration and directions of displacement of elements under load.

**Figure 7:**
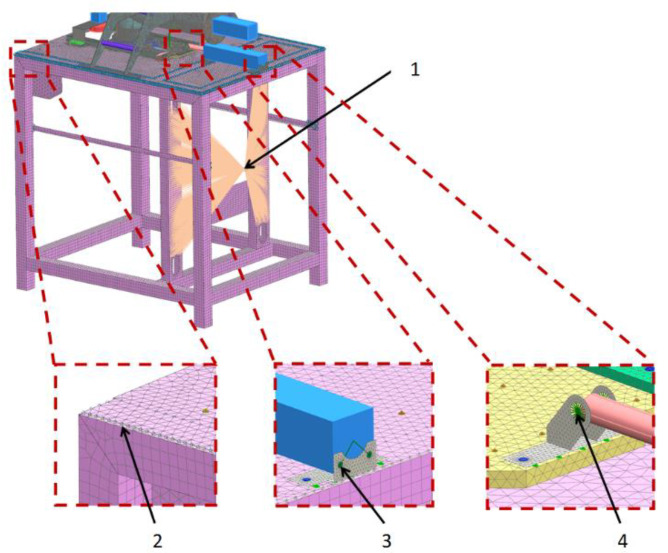
Calculation model of the forming module showing the way of modeling the welds and the actuator connection. 1. The method takes into account the weight of the control box to the mounting bracket: rigid elements are 1D (RBE2) and mass is of the “lumped mass” type. 2. Weld pattern: el. 1D (RBE2). 3. Connection of the actuator with the handle: el. stiff. 4. Connection of the actuator piston rod with the handle: rigid elements (RBE2).

The computational model includes places where there are welds (connections of rectangular sections with sheet elements and connections of the die assembly actuator), as shown in Figure 7. The welds connecting the rectangular sections with sheet metal elements were modeled with rigid 1D elements (RBE2), while the sections were modeled with 2D elements. The welds allowed for the correct transfer of loads from the sheet elements to the sections. The figure also shows the method taking into account the weight of the control box mounted to the support frame (the weight of the box with all accessories was assumed to be 50 kg).

**Figure 8:**
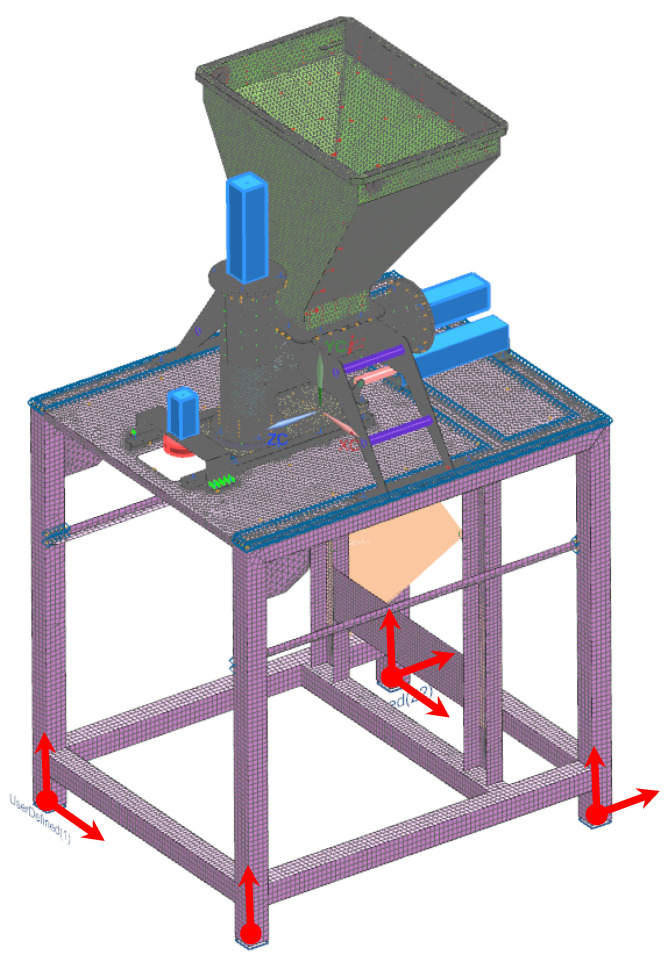
Location of degrees-of-freedom distribution: red vectors indicate directions along which movement is restricted.

The computational model of the molding module was supported as shown in Figure 8. In actual operating conditions, the device assembly was not bolted to the ground. Thus, the degrees of freedom on one leg were distributed in all directions, while on the opposite leg they were distributed vertically and longitudinally, and for the other legs, only in the vertical direction.

**Figure 9:**
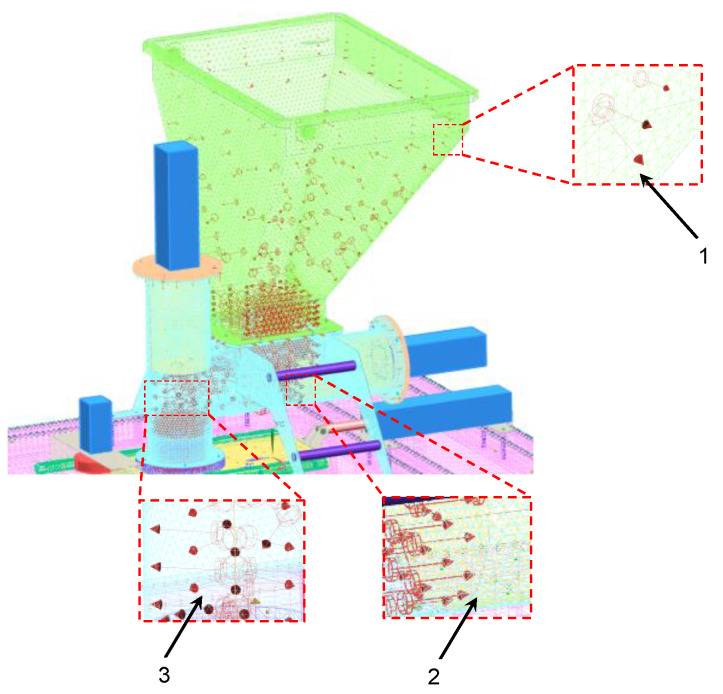
Load application site: charge pressure on the side walls of the technological tank, on the side wall of the piston, and on the side walls of the dispensers. 1. Pressure forces of the vegetable mix on the side walls of the technological tank, 2. Pressure forces of the vegetable mix on the side wall of the piston, 3. Pressure forces of the vegetable mix on the side walls of the dispensers.

The loading of the computational model was carried out as shown in Figure 9, based on the pressure forces of the vegetable mix on the side walls of the technological tank, the horizontal and vertical dispenser, and the pusher walls. Therefore, the load was treated as hydrostatic pressure, where the interaction forces change with height (depth). The density of the pressed mixture was experimentally determined in the laboratory of the Łukasiewicz Research Network–IIAE as 1100 kg·m^−3^. 

**Figure 10:**
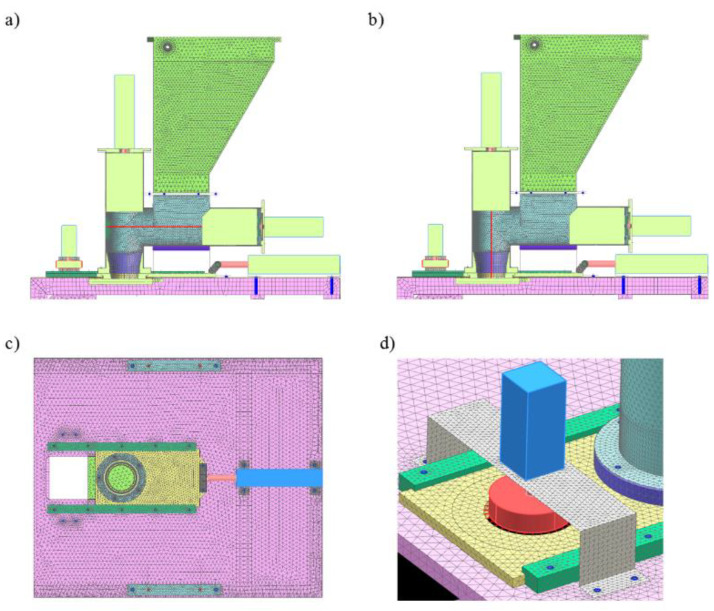
Load cases including forces from pneumatic actuators in undesirable situations: (**a**) blockage of the pusher of the horizontal dispenser; (**b**) blockage of the pusher of the vertical dispenser; (**c**) blockage of the die assembly in the runners; (**d**) accidental collision of the ejector with the die assembly.

The state of effort of the forming module structure was examined in terms of loading with the forces of pneumatic cylinders in undesirable situations, assuming that the horizontal and vertical dispensers were filled with a concentrated vegetable mixture. The following cases were analyzed:

- Blockage of the pusher of the horizontal dispenser. The pusher is pushed by the actuator, and the large amount of mixed vegetable mix prevents it from shifting in the sleeve (Figure 10a). The red line shown in the figure shows that, as a result of the pusher action, the vegetable mix is pressed against the wall of the vertical dispenser (RBE2 rigid element). In this simplification of the calculation model, the compressibility of the mixture was omitted.
- Blockage of the pusher of the vertical dispenser (Figure 10b).
- Locking of the die assembly in the runners (Figure 10c).
- Accidental collision of the ejector with the die assembly (Figure 10d).

## 3. Results and Discussion

**Figure 11:**
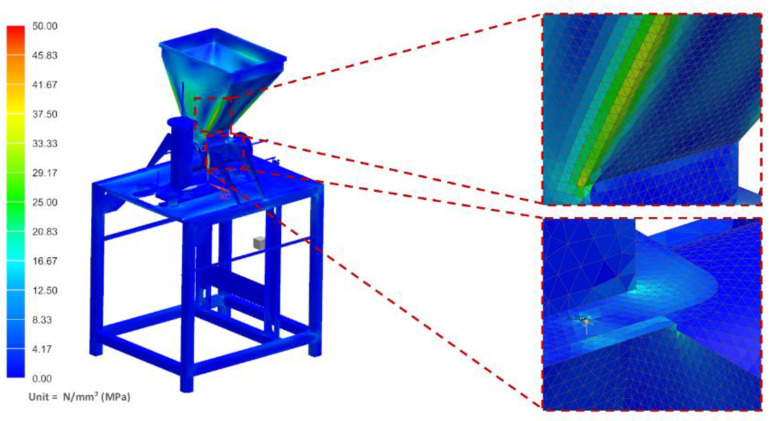
H–M–H stress map of the molding module.

**Figure 12:**
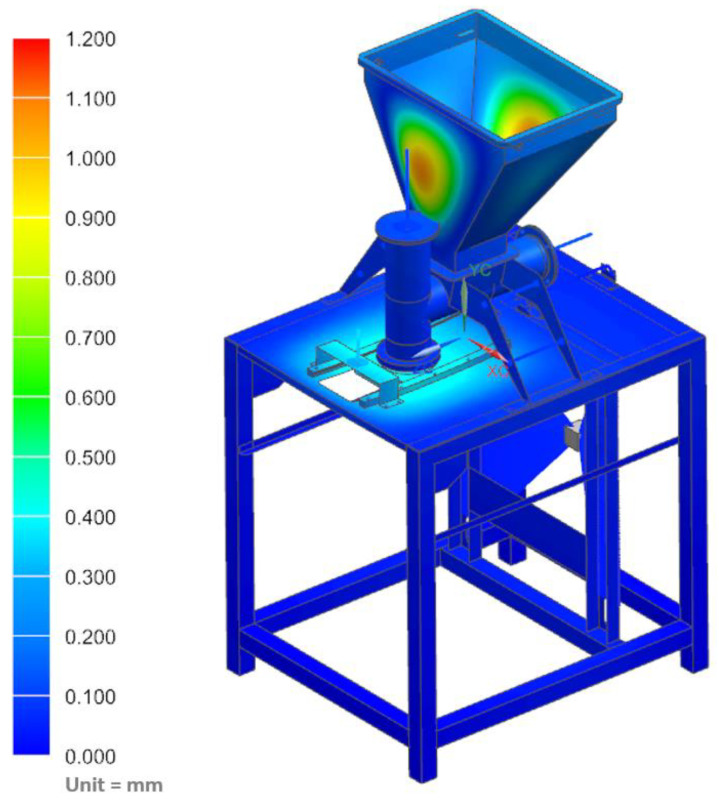
Displacement map of the molding module.

The strength analyses of the forming module for the load of the structure with the weight of the vegetable mixture, considering the pressure on the side walls of the technological tank and dispensers, showed that the maximum stresses occurred in the area of the tank weld and amounted to 34.42 MPa (Figure 11). Moreover, the analyses revealed that the maximum displacement occurred on the side walls of the tank and amounted to 1.13 mm (Figure 12).

In the next part of the analysis, the state of effort of the structure related to the forces generated by pneumatic actuators was examined for specific cases.

### 3.1. Case 1. The Pusher of the Horizontal Dispenser Was Blocked

**Figure 13:**
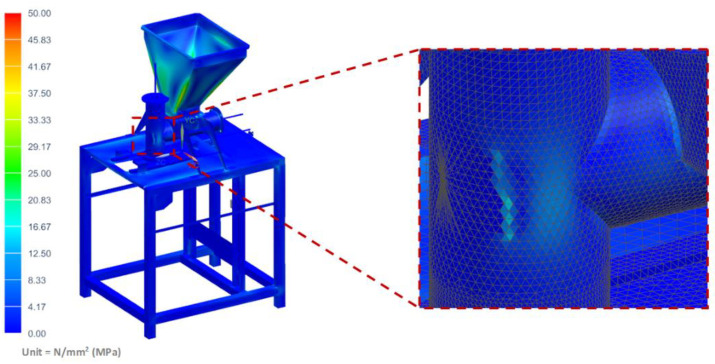
H–M–H stress map of the molding module: pusher of the horizontal dispenser is blocked.

**Figure 14:**
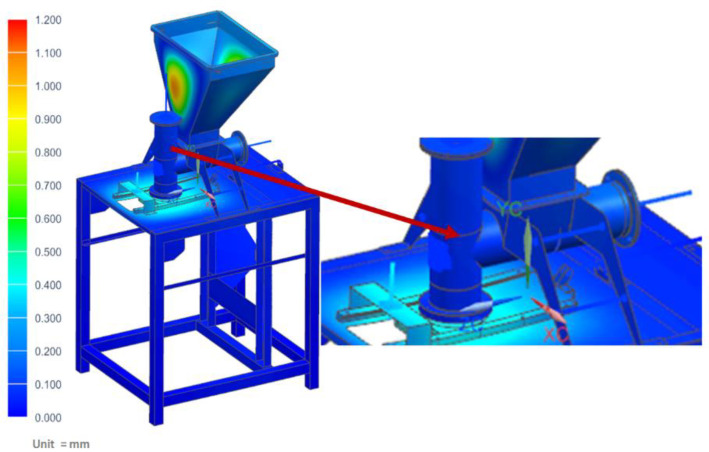
Displacement map of the molding module: pusher of the horizontal dispenser is blocked.

In the computational case assuming that the pusher of the horizontal dispenser was blocked by the vegetable mix accumulated in the chamber and was pressing the mixture with the maximum force generated by the actuator, amounting to 800 N, the maximum stress on the housing of the actuator assembly (specified in Figure 13) was determined as 16.02 MPa. In contrast, the displacement of the forming module was 1.14 mm or less (Figure 14).

### 3.2. Case 2. The Pusher of the Vertical Dispenser Was Blocked

**Figure 15:**
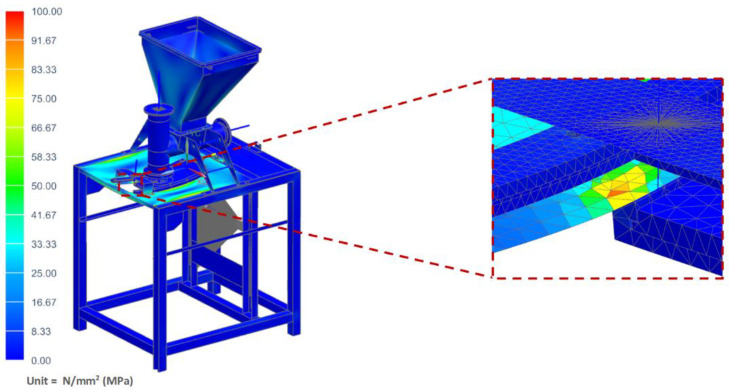
H–M–H stress map of the molding module: pusher of the vertical dispenser is blocked.

**Figure 16:**
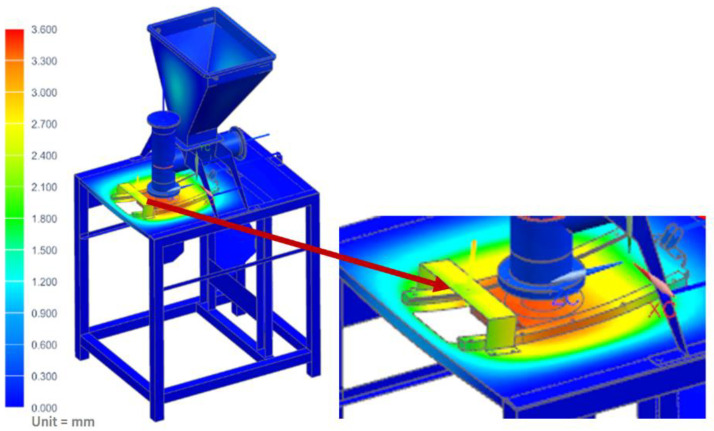
Displacement map of the molding module: pusher of the vertical dispenser is blocked.

In this case, it was assumed that the vegetable mix was pressed against the table, and that during operation, the pusher of the vertical dispenser was blocked against the vegetable mix accumulated in the chamber and pressing against the mixture with the maximum force generated by the actuator of 800 N. The maximum stress of the table where the technological tank with a set of actuators was located (shown in Figure 15) was determined as 97.31 MPa. However, the maximum displacement on the table was only 3.45 mm (Figure 16).

### 3.3. Case 3. The Die Assembly Was Locked in the Runners

**Figure 17:**
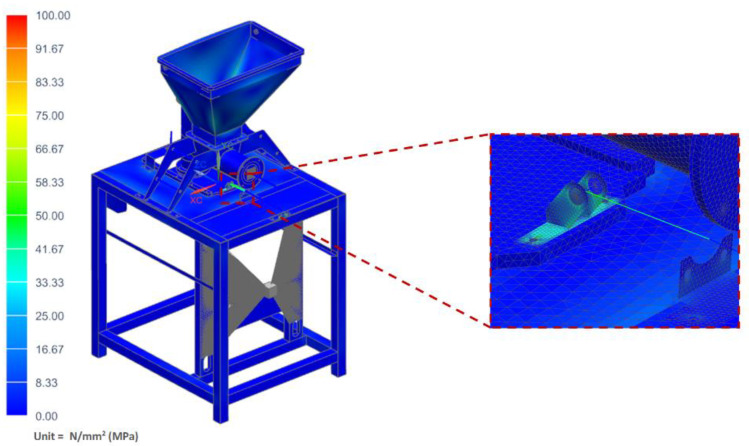
H–M–H stress map of the molding module: die assembly is locked in the runners.

**Figure 18:**
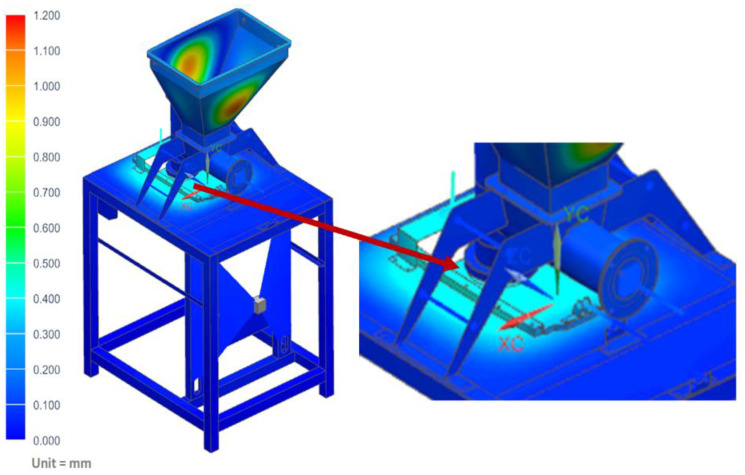
Displacement map of the molding module: die assembly is locked in the runners.

This case assumed the die assembly was locked in the runners when trying to move the die, with the force generated by the actuator amounting to 800 N. The maximum stress at the place indicated in Figure 17 was 55.30 MPa. However, the maximum displacement on the table was only 0.44 mm (Figure 18).

### 3.4. Case 4. The Ejector Collided Randomly with the Die Assembly

**Figure 19:**
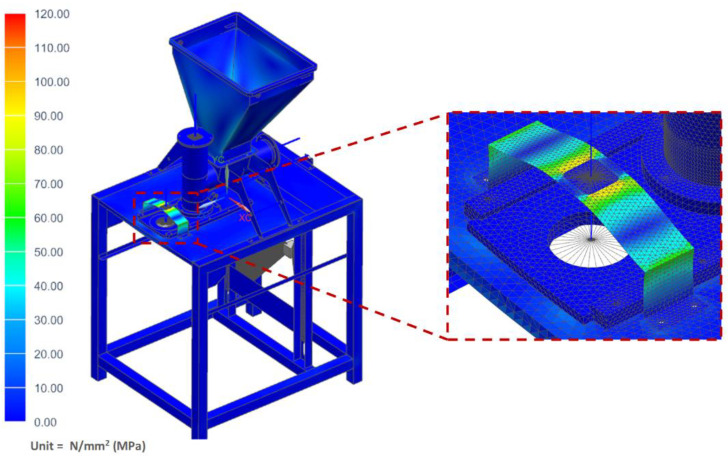
H–M–H stress map of the molding module: ejector collides with the die assembly.

**Figure 20:**
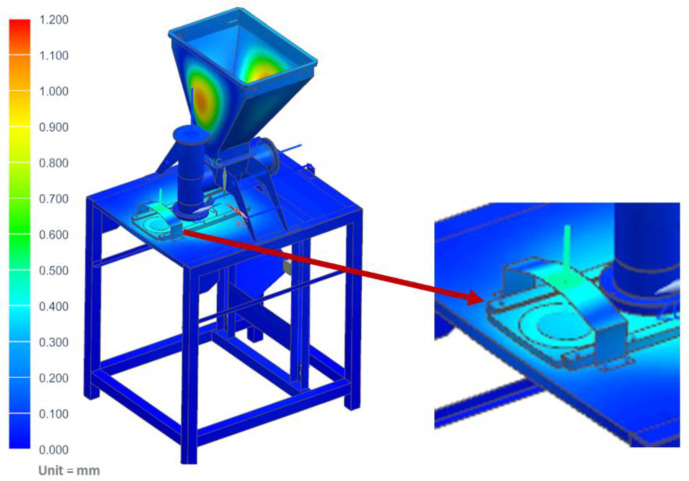
Displacement map of the molding module: ejector collides with the die assembly.

In this case, it was assumed that the ejector randomly collided with the die assembly, with the force generated by the 300 N actuator. The maximum stress in the place specified in Figure 19 was 108.55 MPa. However, the maximum displacement on the table was only 0.51 mm (Figure 20).

The strength analyses carried out using FEM showed that the molding module met the short-term strength criteria. The static calculations of the forming module performed for the loads arising during normal operation in the instantaneous range and for the loads arising during undesirable situations (i.e., the four described cases) showed that the values of maximum equivalent stresses (σ_red_) were 16.02, 97.31, 55.30, and 108.55 MPa for case 1, case 2, case 3, and case 4, respectively. The values of maximum deformation of the structure were 1.14, 3.45, 0.44, and 0.51 mm, respectively. It was found that the highest stresses arose during the collision of the ejector with the die assembly (case 4), with a maximum value of 108.55 MPa. However, this value does not exceed the safety factors, according to which the equivalent stress is 1.1 of the yield strength of the material (σ_red_ < 207 MPa) used for the construction. It was also found that the values of stresses estimated for welds did not exceed the safety index, where the equivalent stress is equal to 1.14 of the material yield strength (σ_red_ < 138 MPa). The maximum value of the stress in the area of the tank weld was 34.42 MPa. 

Table 1 shows the values of equivalent stresses and displacements for individual load cases.

When the pusher of the vertical dispenser was blocked and the vegetable mix was pressed against the table, there was a displacement within the elastic range of about 3.5 mm. As the permissible stress values were not exceeded, it was confirmed that deformation would not affect the strength of the structure. Commonly, in mechanical constructions, the acceptable value of displacement strongly depends on the requirements of the technological process being carried out. The quality of the final product to be obtained determines the level of acceptability of the results. Injection mold designers must meet strict requirements for shape deviations. Insufficient mold stiffness may lead to products that do not comply with the nominal dimensions. For example, in the case of plastic products (remote control housings, telephone housings, etc.), the injection mold stiffness standards require that the maximum value of deformation in the elastic range does not exceed the specified dimensional tolerance of the product. For elements made of ABS, the deformation should be in the range of 0.05–0.08 mm [36]. The injection mold must therefore be sufficiently stiff and the thermal processes must be adjusted so that the displacement due to process loads is within the prescribed range. On the other hand, for tanks subjected to pressure and thermal loads, displacements of the outer plating of a few millimeters in the elastic range are permissible. The layout does not have to be very rigid as this does not affect the quality of the final product. In the structural analysis of food machines, special attention is paid to ensuring that the elastic deformations of the structure do not affect the course of the technological process or reduce the quality of the products obtained. Thus, the value of the table’s elastic displacement, determined in the present analyzed case as amounting to 3.5 mm, is acceptable, because it has no effect on the strength of the structure, nor probably on the product obtained in the technological process. The adopted design assumptions were found to be correct, and therefore they can be used to build a prototype and perform detailed tests in real conditions. Based on the prototype tests, it will be possible to check whether the quality of the finished product does, in fact, change as a result of a displacement of 3.5 mm. Checking the quality of the finished product will be the subject of future research. In addition, experimental studies will be carried out on a real object, together with a comparative analysis of the results obtained with the simulation results. 

## 4. Conclusions

The performed strength analyses of the forming module allowed the formulation of the following conclusions:

- The design of the device corresponds to the assumptions of the technological process, which consists of the processing of frozen vegetable outgrades;
- Strength analyses on a virtual CAD 3D model, allowed an innovative machine that reduces food waste to be designed;
- The results of strength analyses confirmed that the permissible stress levels were not exceeded in the molding module.

Further research work will focus on experimental research carried out to validate the results of the computer simulations using virtual 3D CAD models.

## Figures and Tables

**Table 1:** List of equivalent stresses and displacements.

	Stress (MPa)	Displacement (mm)	Safety Index (Stress)
Loading the structure with the vegetable mixture	34.42	1.13	6.01
Case 1	16.02	1.14	12.92
Case 2	97.31	3.45	2.13
Case 3	55.30	0.44	3.74
Case 4	108.55	0.51	1.91

## Data Availability

Correspondence and requests for materials should be addressed to Ł.I.
